# Amadis: A Comprehensive Database for Association Between Microbiota and Disease

**DOI:** 10.3389/fphys.2021.697059

**Published:** 2021-07-14

**Authors:** Long Li, Qingxu Jing, Sen Yan, Xuxu Liu, Yuanyuan Sun, Defu Zhu, Dawei Wang, Chenjun Hao, Dongbo Xue

**Affiliations:** ^1^Department of General Surgery, The First Affiliated Hospital of Harbin Medical University, Harbin, China; ^2^Key Laboratory of Hepatosplenic Surgery, Ministry of Education, The First Affiliated Hospital of Harbin Medical University, Harbin, China; ^3^Department of Cardiology, The First Affiliated Hospital of Harbin Medical University, Harbin, China; ^4^Family Medicine General Practice Clinic, The University of Hong Kong-Shenzhen Hospital, Shenzhen, China

**Keywords:** gut microbiota, human diseases, network, database, bioinformatics

## Abstract

The human gastrointestinal tract represents a symbiotic bioreactor that can mediate the interaction of the human host. The deployment and integration of multi-omics technologies have depicted a more complete image of the functions performed by microbial organisms. In addition, a large amount of data has been generated in a short time. However, researchers struggling to keep track of these mountains of information need a way to conveniently gain a comprehensive understanding of the relationship between microbiota and human diseases. To tackle this issue, we developed Amadis (http://gift2disease.net/GIFTED), a manually curated database that provides experimentally supported microbiota-disease associations and a dynamic network construction method. The current version of the Amadis database documents 20167 associations between 221 human diseases and 774 gut microbes across 17 species, curated from more than 1000 articles. By using the curated data, users can freely select and combine modules to obtain a specific microbe-based human disease network. Additionally, Amadis provides a user-friendly interface for browsing, searching and downloading. We hope it can serve as a useful and valuable resource for researchers exploring the associations between gastrointestinal microbiota and human diseases.

## Key Points

• Manually curated bioactive associations between gut microbiota and diseases are provided.
• Amadis provides custom network diagram construction function.
• Facilitation of exploring the bacterial community interactions during diseases is available.
• Facilitation of exploring the mechanism of gut microbes–diseases association from the “microbiota-gut-organ” axis perspectives is provided.
• Amadis provides a user-friendly website for browsing, searching and downloading the results.

## Introduction

The human gut microbiome is a complex ecosystem (Weersma et al., 2020). Shaped by long-term coevolution, host-microbiota associations have been cultivated into mutually beneficial relationships. The microbiota provides hosts with genetic, transcriptomic and metabolic attributes that manipulate host biology in both beneficial and harmful ways. Over the past decades, it has been illustrated that microbial communities are important for human and environmental health. Members of the gut microbiota interact with their hosts, establishing advantageous relationships and influencing health throughout the life of the host.

Overwhelming evidence from sequence-based studies indicates that variations and changes in the composition of the gut microbiota influence normal human physiology and contribute to diseases ranging from Alzheimer’s disease to diabetes mellitus. To gain a more comprehensive image of the functions performed by complex microbial communities (Lloyd-Price et al., 2019), it has become prevalent to deploy and integrate multi-omics (metatranscriptomics, metaproteomics, and metabolomics) technologies to reveal essential messages and the secrets hidden behind them (Garrett, 2020). Information gained by combining these different -omics measurements is leading to improved diagnostics, automated drug discoveries, optimized culture conditions for uncharacterized microbes and a better understanding of the functional processes occurring in the system (Jansson and Baker, 2016).

As a result, it has been experimentally validated that the gut microbiota is associated with a wide range of human diseases and communicates with distant organs, such as the lung, heart, liver, and kidney. The concept of the “microbiota-gut-organ” axis has emerged from a recent discovery and has risen as a major topic of research interest in biology. For example, clinicians are well aware of the benefit of rifaximin and rifaximin plus probiotics in the treatment of hepatic encephalopathy (Zuo et al., 2017). Alzheimer’s disease (Paley et al., 2018), Parkinson’s disease (Lai et al., 2018) and refractory epilepsy (Olson et al., 2018) are associated with the relative abundance of various bacterial divisions. The gut microbiota communicates with the CNS through neural (Li et al., 2018), endocrine (Benedek et al., 2017) and immune pathways (Amini-Khoei et al., 2019) and thereby influences brain function and behavior. Furthermore, modulation of the gut microbiota may be a tractable strategy for developing novel therapeutics for multiple human diseases. In 2013, Elsvan Nood et al. studied the effect of duodenal infusion of donor feces in patients with recurrent *Clostridioides difficile* infection (van Nood et al., 2013). Fecal microbiota transplantation (FMT) has been believed to be a reliable rescue treatment for recurrent *Clostridioides difficile* infection (R-CDI) with an ∼90% success rate (Poyet et al., 2019). In addition to FMT, the structure and composition of microbial communities could also be reprogrammed by medicines (Shi et al., 2018), probiotics (Qu et al., 2018), bacteriophages (Dong et al., 2020) or other means. With accumulating investigational trials and experiments in new areas, engineering the gut microbiota to treat disease is an emerging concept.

Currently, a robust collection of multi-omics data about gastrointestinal microbiota-disease associations have been generated in a short amount of time due to the rapid development of this field. However, these associations are scattered among many published studies. The large number of articles is troublesome for researchers who want to further explore the relationships between microbiota and diseases from a global view. More recently, researchers have endeavored to develop online repositories for storing and managing microbiota genomes, disease-related gene and protein signatures, metabolite signatures of diseases, and even providing analysis flows, such as the NCBI Taxonomy database (Federhen, 2012), GeneSigDB (Culhane et al., 2012), MetSigDis (Cheng et al., 2019), gutMDisorder (Cheng et al., 2020), and MEtaGenome Atlas (gutMEGA) (Zhang et al., 2021). Given that gut microbiota are in a complex symbiotic environment, only demonstrating relationships focusing on a single species or one disease has limited significance in revealing the role of intestinal microbiota in physiological and pathological processes. Larger datasets and comprehensive analysis functions will better help researchers explore the relationship between microbiota and human diseases. Showing the dysbiosis in various disease conditions is helpful to reveal the complex, interactive nature of trace microbiota changes in CNS diseases. Studying the pathogenicity mechanisms of a certain species of bacteria in a variety of diseases may identify new therapeutic targets. To date, this kind of analysis function has not been provided.

Therefore, we developed Amadis, a manually curated database that documents experimentally supported microbiota-disease associations and provides functional network visualization and topological analysis. The database is freely available at http://www.gift2disease.net/GIFTED. The current version of Amadis documents more than 20,000 associations between human diseases and gut microbes, curated from more than 1,000 articles. Terms of diseases and microbiota were organized according to International Classification of Diseases (ICD-10) (Hirsch et al., 2015), United States National Center for Biotechnology Information (NCBI) Taxonomy and Medical Subject Heading disease categories (MeSH). The microbiota-disease relationships are sorted by the way microorganisms interact with each other and with their hosts, such as the kinds of toxins produced by the microbes (Cougnoux et al., 2016), the microbiota’s effects on host immune cells and their functional states (Feng et al., 2018) and the types of molecular modification (Armand et al., 2019) caused by dysbiosis of gastrointestinal microbiota in host cells. Additionally, we constructed network diagrams to depict the role of the microbiota in important microbiota-gut-organ axis. Amadis also provides a custom network diagram construction function. Users can select information about diseases, bacteria, genes, etc., of interest to build a relationship network. We expect Amadis to serve as a useful and valuable resource for researchers who seek to understand the functions and molecular mechanisms of GI microbiota involved in human diseases.

## Materials and Methods

### Data Collection

The aim of the Amadis database is to provide comprehensive information about experimentally validated associations between gastrointestinal microbiota and human diseases. To achieve this, we obtained detailed information about microbiota-diseases relationships from published research articles. To ensure high-quality data collection, all Amadis entries about gut microbiota and disease were manually extracted from publications in a specific and precise manner as used for HMDD (Huang et al., 2019), Lnc2Cancer (Ning et al., 2016) and NSDNA (Wang et al., 2017). Up to April 2020, more than 4,000 papers potentially related to gut microbiota and human disease were selected by retrieving the PubMed database with a list of keywords. These keywords may be divided into two categories: (1) names of diseases and microbiota, such as Crohn’s disease, non-alcoholic fatty liver disease, obesity, *Escherichia coli*, and *Akkermansia*; and (2) terms about pathogenic mechanisms, such as genotoxins, inflammatory responses and monocyte differentiation. Then, we retrieved the disease and microbiota names, intervention factors, experimental techniques (e.g., microarray, next-generation sequencing, gas chromatography-mass spectrometry), species of experimental animals, experimental samples (cell line, tissue, blood, and feces), expression patterns of dysregulated genes (upregulated and downregulated), dysbiosis of microbiota, hyperlinks to the PubMed database (PMID, publication year, title) and a brief functional description of microbiota from the original studies. Finally, all microbiota-disease associations and entries mentioned above were double-checked by different researchers.

### Nomenclature Standardization and Classification

The current version of Amadis documents massive disease-microbiota data. Diverse descriptions of the microbiota and disease are used in research articles, thus we standardized these names. To better display data and facilitate accessibility of the database, we organized microbiota terminologies based upon the controlled vocabulary of the MeSH disease categories. Moreover, we integrated the raw taxa at the lowest classification level and obtained their high-level names from the NCBI Taxonomy. Then, we used several disease terminology systems, such as disease ontology (DO)^1^, UMLS^2^, and ICD-10^3^, to describe diseases found to be related to microbiota. Diseases were sorted by 8 systems and 23 organs according to ICD-10. Moreover, since studies provide insight into the mechanisms contributing to microbiota-disease relationships, we classified entries by the mechanism, such as the kinds of toxins, the microbiota’s effects on functional state of immune cells and the types of molecular modifications caused by dysbiosis of GI microbiota in host cells.

### Network Development

With the data curated in Amadis, a network can be constructed to visualize the global relationships between diseases, gut microbiota and genes. We integrated the ECharts plugin software (V4.0)^4^ into the Amadis framework to perform network visualization. For inputted diseases, microbiota or genes, Amadis will determine their neighbors and construct a biological network on the web page. In the network, diseases, gut microbiota, and genes are shown as different color nodes. According to the gene regulation pattern, Amadis will also construct different subnetworks in which upregulation is illustrated as red links and downregulation is illustrated as blue links. Amadis will perform a new search by clicking on each node in the network.

### Database Framework and Web Interface

A user-friendly web interface was developed to present Amadis. All data were organized and managed by the MySQL data server. The web interface for browsing and searching was implemented by Java Server Pages (JSP). Apache Tomcat software (v7.0) was used for the http server.

## Results

### Database Content

**FIGURE 1:**
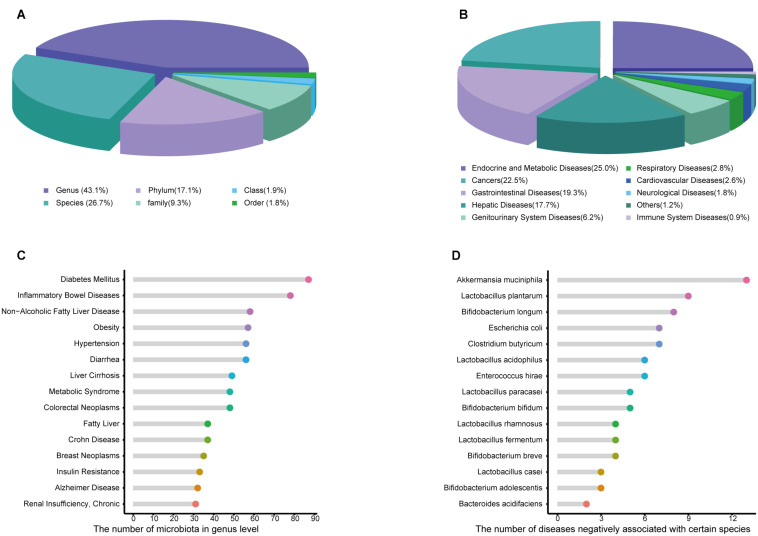
**(A)** The distribution of gut microbiota in Human among different taxa. **(B)** Distribution of relationships based on diseases. **(C)** The number of microbiota related to single disease in genus level. **(D)** The number of diseases negatively associated with a single microbiota in species level.

Amadis offers detailed information about each entry, such as the names of the microbiota and disease, the up-level names of microbiota, the target gene involved, the interventional factor, the evidence of the relationship, the simple type, the specific mechanisms, the change in microbiota compared with healthy controls, the metabolite type, the methods used in the studies, a summary of conclusions and the PMID of the corresponding literature reference. Since 16S rRNA sequencing provides genus-level information and metagenomic approaches can provide species-level information, Amadis integrates seven classification levels of information (Figure 1A), distributed in phylum (17.1%), class (1.9%), order (1.8%), family (9.3%), genus (43.1%), and species (26.7%). The genus and species levels occupy most of the data. Exploration of disease data indicated the significant effects of microbiota (Figure 1B). As shown in Figure 1C, compared with healthy donors, gut microbiota abundances in patients with diabetes mellitus changed dramatically, since it was associated with the greatest number of microbes at the genus level. In the data with experimental evidence, *Akkermansia muciniphila* arrested the progression of 13 diseases, showing potential benefits as a probiotic (Figure 1D). Among the data at the species level recorded by Amadis, most microbiota only changed in one disease (Supplementary Figure 1). These microbiota may therefore be used as biomarkers in the data with experimental evidence.

### Network Visualization and Topological Analysis

To allow users to easily explore the relationship between gut microbiota and diseases on their own, we tried to provide the function of building a network diagram independently on the network page. To achieve this, we developed a network visualization interface using ECharts plugin software, where the global three-membered network and direct interactions of a certain microbiota, gene, or disease can be viewed (see Help page of Amadis for details). Users can select a variety of bacteria, diseases, and gene names included in the database and build a relationship network diagram based on the information provided in the database.

At present, it is accepted that a complex bidirectional communication system exists between the gastrointestinal tract and remote organ diseases. The term “microbiota-gut-organ axis” demonstrates the pivotal role of gut microbiota in maintaining local and systemic homeostasis. To better demonstrate the effect of the intestinal microbiota on these diseases and to discover “hub-microbiota” that may play a central role in diseases, we used the data obtained to construct comprehensive networks of bacteria, genetic information and all diseases in a single axis, such as the microbiota-gut-brain axis and the microbiota-gut-liver axis.

### Database Utility

#### Homepage

**FIGURE 2:**
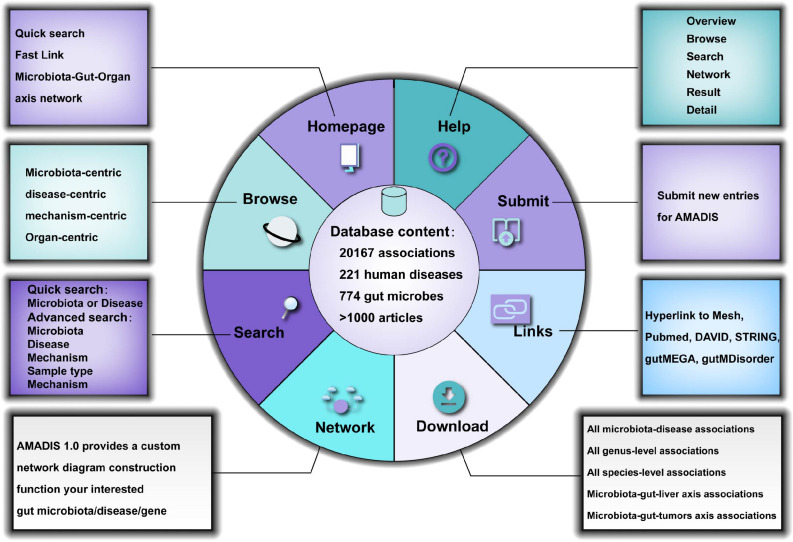
Content and interface of Amadis.

Amadis provides a user-friendly web interface for an easy database query (Figure 2). To explore the Amadis database content rapidly, we have provided a quick search option on the home page of Amadis. Meanwhile, a comprehensive network analysis function was also provided. Users can obtain as many as 11 comprehensive analysis networks and gain a more comprehensive image of the diseases, gut microbiota, and genes involved in these networks.

### Browsing

To clearly display the manually curated data, we developed a powerful browsing tool. There are five logical categories: microbiota, disease, organ, system and mechanism in the navigation bar on the left. Each category contains subcategories. Users can browse relevant entries in Amadis by clicking any drop-down menus or their submenus. Using “*Bacteroides*” as an example, the page of this entry displays the related disease, the sample type, the -omics technology, and the mechanism involved. Each entry contains detailed information, including the disease and microbiota names, the species involved, the association between the disease and microbiota (e.g., Microbiota promote disease progression and Microbiota inhibit disease progression), the experimental methods used (e.g., Transcriptomics and Real-time PCR), the detected tissue (e.g., brain and blood), the associated factors (e.g., levodopa and Interferon-Beta), the associated genes (e.g., MCAD and Fas), and a detailed description and corresponding literature (PMID and publication year).

#### Search Function

On the search page, four search fields are present that may be used together or separately: (1) microbiota, (2) disease, (3) sample type, and (4) mechanism. A user can query the database using standardized keywords, e.g., microbiota name, disease name, or sample type in the corresponding search fields. Amadis also offers a fuzzy search engine. The fuzzy search function allows users to retrieve entries by the name of a microbiota species, a disease or a sample type even when the query name is not perfectly clear. Once a certain query name is received, the system searches in the corresponding data field for terminology that contains the query words. The matching terminologies are listed as multiple hits in the pop-up result page. From these retrieved results, users can manually check to obtain the exact entry of interest through relevance to the query term.

### Network Construction

In the lower right part of the homepage, we constructed multiple comprehensive analysis networks of the microbiota-gut-organ axis, such as the microbiota-gut-liver axis and microbiota-gut-brain axis. These network diagrams comprehensively demonstrate the role of microbiota in diseases of distant organs and provide reference materials for selecting research directions. On the network page, we provide a dynamic network construction method and allow users to freely select and combine modules to obtain their own network construction.

Users can select one or more bacterial species, diseases, and/or genes of interest to add to the column to be analyzed on the right. The information in the column can be deleted one by one or cleared all at once. After selecting the molecule, the user can click submit to start network construction and visualization. Clicking on a molecule in the network diagram will search for this molecule and display all entries.

#### Submit and Update

Amadis invites users to submit associations that are not documented in the database on the “Submit” page. To maintain the integrity of the database, we will conduct manual verification of the original publication(s) for data validation upon each submission from nonaffiliated researchers. Once approved by the review committee, the novel associations will be available to the public in the updated version.

#### Download and Help

As a publicly released scientific database, Amadis allows users to download all the obtained data on the “Download” page. In addition, detailed usage and guidelines of the database are available on the “Help” page.

## Discussion and Conclusion

A growing number of people adhere to the idea that “we are what we eat.” Nevertheless, for decades, investigators and clinicians have been trying to reveal the relationship between intestinal microbiota and disease and to improve the diagnosis and treatment of disease. Researchers believe that some diseases are fundamentally not only genetic but also microbial diseases. However, accumulating experimentally supported associations are scattered in thousands of published studies. To provide a comprehensive resource of the functions of microbiota in human disease, several outstanding online repositories have been developed for storing microbiota-related data (van Nood et al., 2013; Benedek et al., 2017; Amini-Khoei et al., 2019).

In this study, we developed Amadis, a manually curated database that documents associations between human diseases and gut microbes, to better show the relationship between the gut microbiota and human diseases, to make full use of the massive amount of data accumulated in recent years, and to assist researchers in seeking novel insights. The database we developed not only obtains data from more than 1,000 articles but also conducts comprehensive analyses and constructs visual networks.

Serving as a useful and convenient resource, researchers could find some important implications behind a large, complex and integrated resource. By analyzing the network of the microbiota-gut-tumor axis, we found that *A. muciniphila* has an inhibitory effect on the development of six different tumors. *Akkermansia muciniphila* components, which are considered prebiotics, may be a new way to treat tumors. At the same time, studying the mechanism *A. muciniphila* uses to inhibit tumor progression may reveal new tumor treatment targets.

Among the dysregulated genes, there are several members of the CXCL family. By mining TCGA and Oncomine data, we found that the differences in the expression of CXCL family members in colorectal cancer, breast cancer and pancreatic cancer were remarkable. There were also significant differences in overall survival (OS) time and disease-free survival (DFS) time among people with different expression levels of CXCL family members. The abnormal expression of CXCL family members in a variety of tumors and their close relationship with OS and DFS time reveal the important role of this family in tumorigenesis and development. Moreover, the CXCL family is closely related to microbiota and may serve as a bridge between microbiota and diseases, especially cancers. Future research may clarify the relationship between the microbiota-CXCL family-diseases axis.

There are studies in the literature reporting that patients with rheumatoid arthritis (RA) have a significantly higher risk of coronary heart disease (CHD). To explore the role of microbiota in this situation, we can use the data documented in the database to construct a network of these two diseases to find microbiota whose abundance changes in both RA patients and CHD patients. By using the network analysis function, we found that the abundance of Blautia, Dorea, and Prevotella changed in both RA patients and CHD patients. These three genera may be involved in mediating the pathological process of CHD in patients with RA.

Overall, Amadis not only provides more than 20,000 manually curated microbiota associations with multi-omics data and experimental support but also offers global insights into microbiota functions in human diseases. Since it is the manually curated repository for annotating the function of gut microbiota, we believe that Amadis will serve as a useful resource for decrypting mechanisms, improving the diagnosis and treatment of human disease.

## Future Development

The Amadis database represents one of the first steps in this project. Further extensions are under way. We will update the repository with experimentally supported association data every 2 months. As stated in the “data collection and database content” section, the microbiota–disease relationships documented in the current version were collected by indexing, cataloging, and searching of biomedical and health-related information on PubMed with a list of keywords referring to MeSH and the NLM-controlled vocabulary thesaurus. Furthermore, text-mining tools will be adopted to help us retrieve PubMed abstracts that potentially describe further microbiota-disease relationships. In addition, we are developing multi-omics profile- and interacting partner-based methods to predict novel microbiota-disease associations and expect to integrate these methods into the database in the future. These strategies will make the cataloging and searching processes more efficient and data content more comprehensive.

## Data Availability Statement

The original contributions presented in the study are included in the article/Supplementary Material, further inquiries can be directed to the corresponding author/s.

## Author Contributions

LL: conceptualization, writing – original draft preparation, and writing – review and editing. QJ: methodology. SY: formal analysis. XL, DZ, YS, and CH: investigation. DW: supervision. DX: project administration. DX and LL: funding acquisition. All authors have read and agreed to the published version of the manuscript.

## Conflict of Interest

The authors declare that the research was conducted in the absence of any commercial or financial relationships that could be construed as a potential conflict of interest.

## Footnotes

1 http://disease-ontology.org

2 http://umlsks.nlm.nih.gov

3 http://www.cdc.gov/nchs

4 http://echarts.apache.org/
