# Supplementary figures and images for: Amadis: A Comprehensive Database for Association Between Microbiota and Disease
Source: Front Physiol. 2021 Jul 14;12:697059. doi: 10.3389/fphys.2021.697059 (PMC8317061; doi:10.3389/fphys.2021.697059)

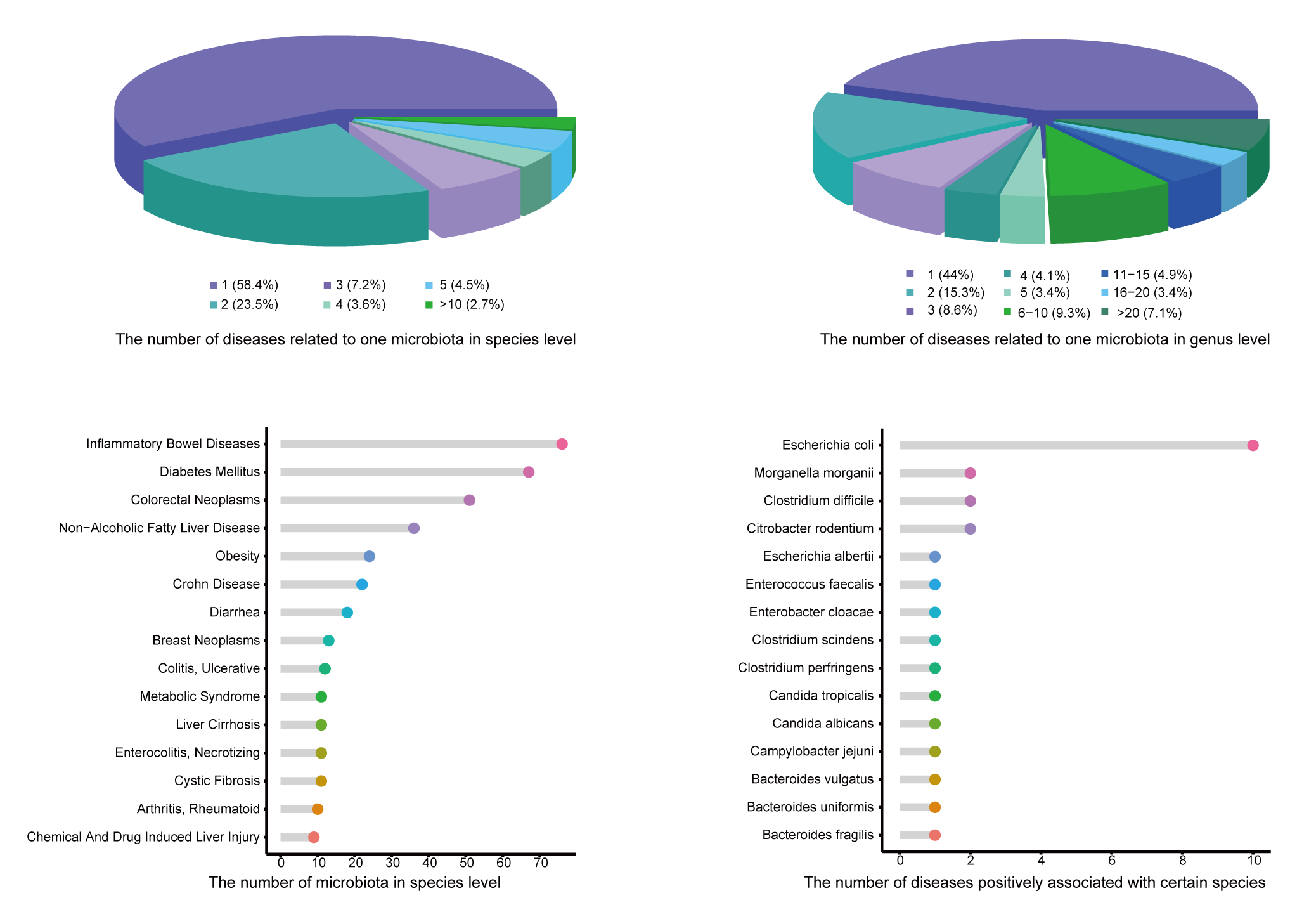

Supplement: Supplementary file 1 [file Image_1.TIF]
